# Supplementary material for: A multi-population phenome-wide association study of genetically-predicted height in the Million Veteran Program
Source: PLoS Genet. 2022 Jun 2;18(6):e1010193. doi: 10.1371/journal.pgen.1010193 (PMC9162317; doi:10.1371/journal.pgen.1010193)
Supplement: S1 Text — Fig A. Associations of selected traits with genetically-predicted height after stratifying by diabetes status. Odds ratio (OR) and 95% confidence intervals shown for associations of the indicated traits with genetically-predicted height in all participants (purple), those without diabetes (green), and those with diabetes (yellow). P-values from test of heterogeneity between strata shown to the right. (PDF) [file pgen.1010193.s001.pdf]

A multi-population phenome-wide association study of genetically-predicted height in the Million Veteran Program

Sridharan Raghavan, Jie Huang, Catherine Tcheandjieu, Jennifer E. Huffman, Elizabeth Litkowski, Chang Liu, Yuk-Lam A. Ho, Haley Hunter-Zinck, Hongyu Zhao, Eirini Marouli, Kari E. North, the VA Million Veteran Program, Ethan Lange, Leslie A. Lange, Benjamin F. Voight, J. Michael Gaziano, Saiju Pyarajan, Elizabeth R. Hauser, Philip S. Tsao, Peter W. F. Wilson, Kyong-Mi Chang, Kelly Cho, Christopher J. O'Donnell, Yan V. Sun, Themistocles L. Assimes

S1 Text. Effect of diabetes mellitus status on genetically-predicted height associations with neurologic, dermatologic, and infectious diabetes complications.

S1 Text, Fig A. Associations of selected traits with genetically-predicted height after stratifying by diabetes status.

Effect of diabetes mellitus status on genetically-predicted height associations with neurologic, dermatologic, and infectious diabetes complications

In the MR-PheWAS, we found associations of genetically-predicted height with phecodes for several peripheral neuropathy conditions, including diabetic neuropathy (OR 1.18 [95% CI 1.12, 1.24] per SD increase in height). However, genetically-predicted height was only modestly associated with diabetes itself with this association not exceeding the phenome-wide significance threshold (OR 0.97, $p=1.7\times10^{-2}$ for all diabetes mellitus; OR 1.05, $p=8.7\times10^{-2}$ for Type 1 diabetes; and OR 0.97, $p=1.3\times10^{-2}$ for Type 2 diabetes in non-Hispanic White individuals). Given the high prevalence of diabetes mellitus among Veterans, we wanted to evaluate whether some of the traits associated with genetically-predicted height might be attributed to miscoding related to diabetes status[1-3], or if diabetes status might modify the association of genetically-predicted height with these traits. Accordingly, we tested associations of these selected traits after stratifying by diabetes mellitus status. The association of genetically-predicted height with hereditary and idiopathic peripheral neuropathy (phecode 356) did not vary by diabetes mellitus status (**Fig A in S1 Text**, heterogeneity $p=0.5$). We also noted genetically-predicted height associations with several infectious and dermatologic conditions that are not uncommonly observed in the setting of diabetes and diabetic neuropathy – chronic lower extremity ulcers, osteomyelitis, and superficial cellulitis (**Table 2**, **S3 Table**). The genetically-predicted height association with chronic leg/foot ulcer (phecode 707.2) was comparable in those without and with diabetes mellitus (heterogeneity $p=0.6$; **Fig A in S1 Text**). In contrast, genetically-predicted height associations with osteomyelitis, periostitis and other infections of bone (phecode 710) and with superficial cellulitis and abscess (phecode 681) were stronger in individuals with diabetes mellitus than in those without diabetes mellitus (heterogeneity $p=3.2\times10^{-3}$ and $p=2.4\times10^{-4}$ for phecodes 710 and 681, respectively; **Fig A in S1 Text**).

References

1. US Department of Veterans Affairs, Office of Research & Development. Close to 25 percent of VA patients have diabetes. *https://www.research.va.gov/topics/diabetes.cfm.* 2015;Accessed January 10, 2018.
2. Liu Y, Sayam S, Shao X, et al. Prevalence of and Trends in Diabetes Among Veterans, United States, 2005-2014. *Prev Chronic Dis.* 2017;14:E135.
3. Eibner C, Krull H, Brown KM, et al. Current and Projected Characteristics and Unique Health Care Needs of the Patient Population Served by the Department of Veterans Affairs. *Rand Health Q.* 2016;5(4):13.

Fig A.

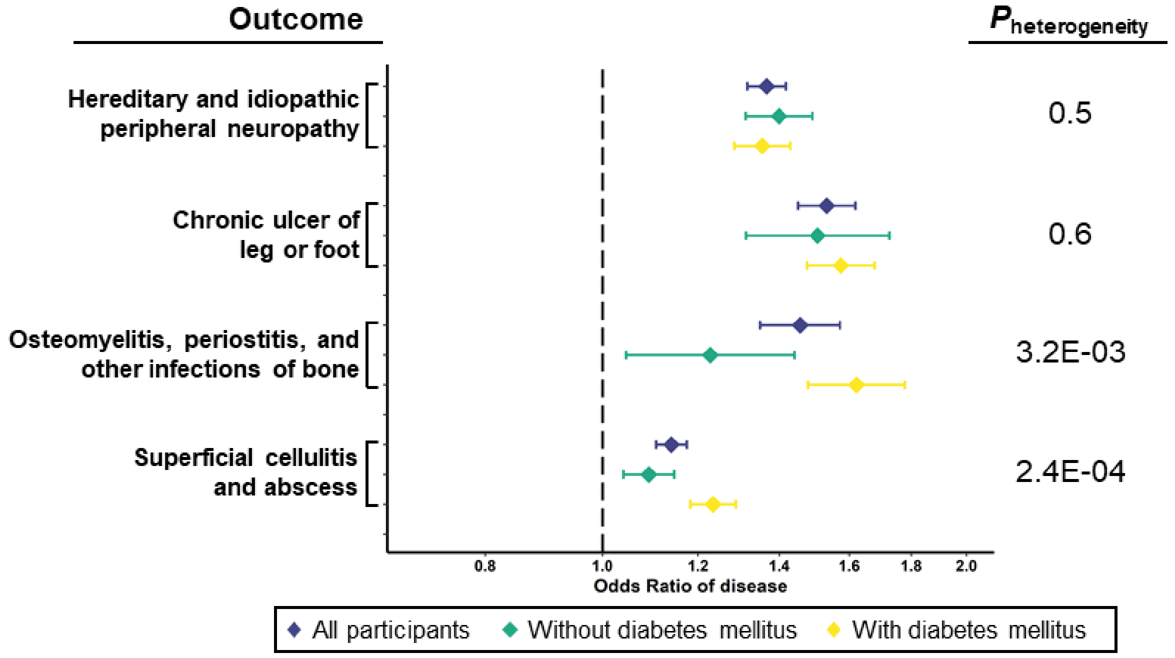

Fig A. Associations of selected traits with genetically-predicted height after stratifying by diabetes status. Odds ratio (OR) and 95% confidence intervals shown for associations of the indicated traits with genetically-predicted height in all participants (purple), those without diabetes (green), and those with diabetes (yellow). P-values from test of heterogeneity between strata shown to the right.
