# Supplementary material for: A multi-population phenome-wide association study of genetically-predicted height in the Million Veteran Program
Source: PLoS Genet. 2022 Jun 2;18(6):e1010193. doi: 10.1371/journal.pgen.1010193 (PMC9162317; doi:10.1371/journal.pgen.1010193)
Supplement: S1 Fig — Comparison of standardized effect sizes (Z’) for associations with height of variants used in height genetic risk score for MR-PheWAS in Yengo et al (Human Molecular Genetics 2018:27(20):3641–3649) and MVP non-Hispanic White (EA, left) and non-Hispanic Black (AA, right) individuals. (PDF) [file pgen.1010193.s002.pdf]

S1 Fig. Effect size comparison of height-associated variants from source European-ancestry GWAS meta-analysis and non-Hispanic White- and non-Hispanic Black MVP participants.

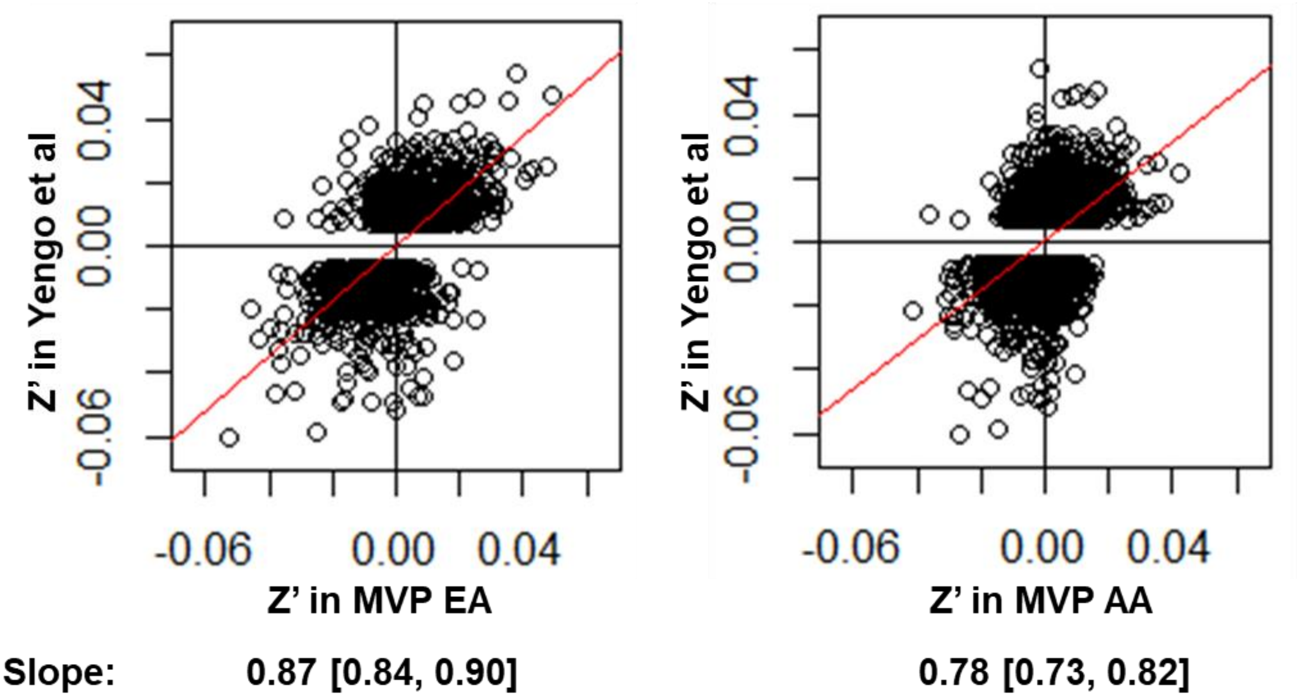

S1 Fig. Comparison of standardized effect sizes (Z') for associations with height of variants used in height genetic risk score for MR-PheWAS in Yengo et al (*Human Molecular Genetics* 2018:27(20):3641-3649) and MVP non-Hispanic White (EA, left) and non-Hispanic Black (AA, right) individuals.
