# Supplementary material for: A multi-population phenome-wide association study of genetically-predicted height in the Million Veteran Program
Source: PLoS Genet. 2022 Jun 2;18(6):e1010193. doi: 10.1371/journal.pgen.1010193 (PMC9162317; doi:10.1371/journal.pgen.1010193)

S2 Fig. Effect size comparison of associations of genetically-predicted height with clinical traits in MR-PheWAS without and with body mass index (BMI) as a covariate in non-Hispanic White (A) and non-Hispanic Black (B) individuals.

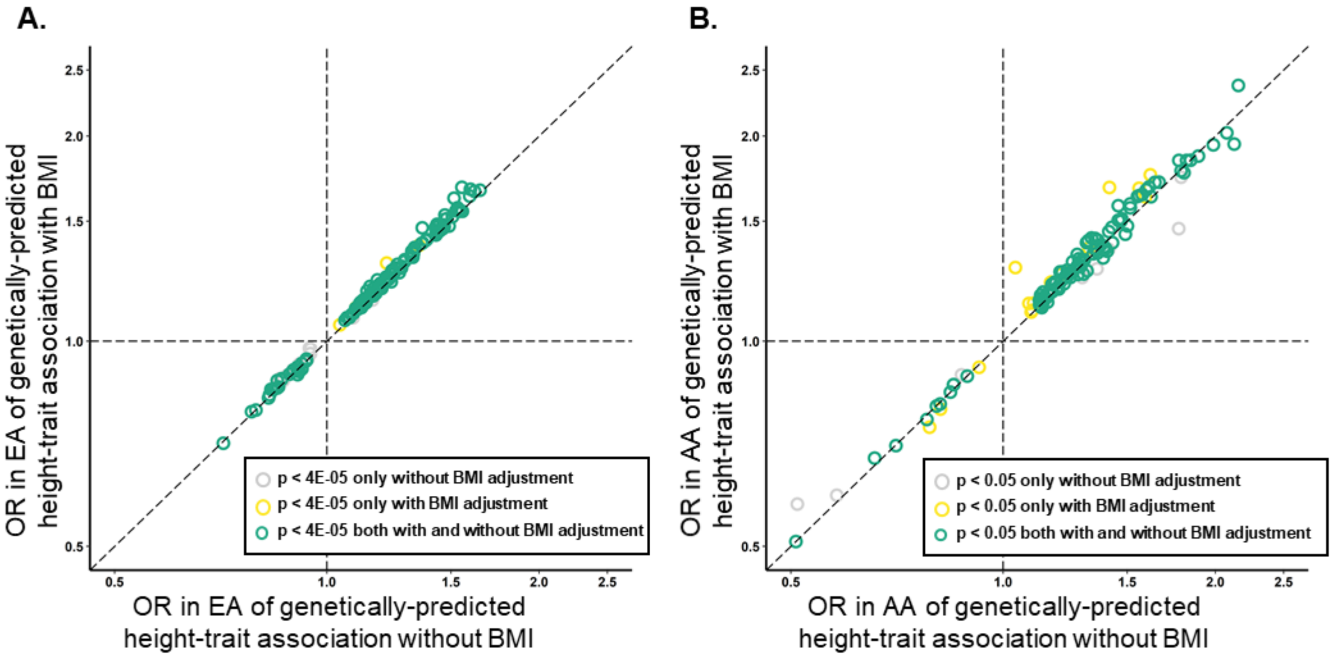

Supplement: S2 Fig — Effect size comparison of associations of genetically-predicted height with clinical traits in MR-PheWAS without and with body mass index (BMI) as a covariate in non-Hispanic White (A) and non-Hispanic Black (B) individuals. (PDF) [file pgen.1010193.s003.pdf]
