# Supplementary material for: A multi-population phenome-wide association study of genetically-predicted height in the Million Veteran Program
Source: PLoS Genet. 2022 Jun 2;18(6):e1010193. doi: 10.1371/journal.pgen.1010193 (PMC9162317; doi:10.1371/journal.pgen.1010193)
Supplement: Attachment — Submitted filename: PLoS_Genetics_Responses_to_reviewers_R1_3-1-2022.docx [file pgen.1010193.s018.docx]

We thank the Editor and Reviewers for their thoughtful review of our manuscript and have attempted to respond to each comment below. Please note that line numbers referenced in the responses to comments correspond to location in the clean revised manuscript, not to the location in the revised manuscript with tracked changes. Edits/insertions responsive to reviewer comments have been included in italics below in the point-by-point response.

**Editor comment:** We have received detailed responses from two expert reviewers, both of which provide thoughtful suggestions for improvement upon revision. These edits will both clarify some outstanding issues and add clarity with some new analyses. Overall, the appraisal of the manuscript is very positive.

**Response to editor:** We appreciate the opportunity to respond to the reviewer comments and to submit a revised manuscript. In addition to text revisions to improve clarity, we have added the following analyses to the revised manuscript:

1. Meta-analysis of MR-PheWAS across race/ethnicity groups.

2. Sex-stratified MR-PheWAS analysis.

3. LD score regression of GWAS results in non-Hispanic White individuals.

**Reviewer Comments to the Authors:
Reviewer #1:** In this study, Raghavan et al. quantify the effect of a polygenic predictor of height on various clinical conditions in a large ancestrally diverse sample from the Million Veteran Program. Overall, they find hundreds of associations between height and various disease, including previously reported signals. This study is an important contribution to the literature, which demonstrates that height is indeed an underappreciated risk factor for many diseases. However, I found that the study did not fully utilise the potential of the dataset and raise below a points that could improve the quality of the study.

**Comment 1.1.** I found the GRS analysis to be the most interesting part of the manuscript. The phenotypic analysis (height vs disease) could have been utilised as a way filter diseases to focus on but the fact that the authors present those analyses in parallel, reduces the interest of the phenotypic analysis alone.

**Response 1.1.** While we appreciate the reviewer’s suggestion, we feel the current organization is important to the narrative of the study. The phenotypic analysis establishes the scope of associations in the MVP data, which may differ from other study samples with different characteristics. Related to this, we feel it is important to establish concordance of the height-trait associations between EA and AA race/ethnicity groups before proceeding to the analysis of genetically-predicted height stratified by race/ethnicity given the limitations of the MR-PheWAS in AA discussed later in the manuscript (reduced sample size, weaker genetic instrument). Retaining the phenome-wide scope for each of the association analyses in each race/ethnicity group was the most unbiased approach in our view. In contrast, filtering based on the height-trait analyses would seem to privilege the race/ethnicity with the larger sample size that also happens to have a better genetic instrument (EA). As this reviewer and reviewer 3 both commented on the manuscript’s organization (though with different suggestions), we have created additional sections in the Results to separate the associations of traits with measured height from associations of traits with genetically-predicted height. Hopefully this improves readability of the manuscript.

**Comment 1.2.** The authors report what seems to be a good concordance of the effect of GRS on disease risk across ancestries. Such an observation calls for a meta-analysis across ancestry groups, which may also increase power.

**Response 1.2.** In response to this suggestion, we have meta-analyzed the MR-PheWAS results in European- and African-American race/ethnicity groups. The meta-analysis did not introduce any new traits among the most highly associated phecodes shown in Table 2. We have added the meta-analysis results to Table 2 and added **Supplemental Table 6** with PheWAS results for the five traits that achieved phenome-wide significance after meta-analysis but were below the significance threshold in EA and AA alone. The following have been added to the text of the manuscript:

Results (lines 224-230): *“Meta-analyzing genetically-predicted height associations from EA and AA individuals identified 141 total traits associated with genetically-predicted height at phenome-wide significance (p<3.6×10^-5^). Of these, five were phenome-wide significant only after meta-analysis and not in either race/ethnicity group alone (****Supplemental Table 6****).* ***Table 2*** *shows the top ten and closely related phecodes associated with genetically-predicted height in EA individuals that achieve at least nominal significance in AA individuals, all of which were phenome-wide significant in the meta-analysis of EA and AA associations.”*

Discussion (lines 329-333): *“**Meta-analysis of results from EA and AA individuals identified five additional traits associated with genetically-predicted height. Two were genitourinary conditions – erectile dysfunction and urinary retention – that can be associated with neuropathy, and a third was a phecode for non-specific skin disorders that may be related to skin infections – consistent with the race/ethnicity stratified results.”*

Methods (lines 546-549): *“Finally, we performed a fixed effects meta-analysis of MR-PheWAS results with genetically-predicted across EA and AA race/ethnicity groups in METAL^55^, applying a significance threshold of p<3.6×10^-5^ corresponding to the threshold for the race/ethnicity group with the larger sample size (EA).”*

**Comment 1.3.** The authors perform a GWAS in a European ancestry sample of the MVP. This analysis is interesting but they authors do not use those results to further improve power to address their main question: the association between height PRS and disease. By meta-analysing Yengo et al. with their MVP-EA GWAS, they could have increased power to detect association in African American.

**Response 1.3.** We appreciate the reviewer’s suggestion but intentionally did not focus on the GWAS portion of the analyses reported in this manuscript for two reasons. First, we have contributed GWAS data to a large international, multi-ancestry height GWAS meta-analysis now available online in pre-print form (Yengo et al, *biorxiv* <https://doi.org/10.1101/2022.01.07.475305>), and a meta-analysis of our data with Yengo et al (2018) would not have matched the scope of the broader consortium. Second, we did not want to risk overfitting when estimating genetically-predicted height, so opted to use GWAS results from an independent sample from the PheWAS sample. Therefore, we did not use the GWAS results from within sample to modify the GRS used for the MR-PheWAS; the same issue would have arisen if we’d performed a meta-analysis with Yengo et al (2018) to improve the GRS. We have now added a citation to the Yengo et al *bioRxiv* paper in the Discussion of limitations and need for larger multi-ancestry GWAS of height.

**Comment 1.4.** The authors rightly underline that an association between height GRS and disease may not necessarily reflect causality but possibly pleiotropy. Nevertheless, as I reader I’d like to know how many (statistically) independent associations are reported here. The grouping in terms of broad disease category partly addresses my question but it still possible that pleiotropic effect exist beyond phecodes. For example, is the effect of GRS on Atrial fibrillation independent of that on varicose veins?
**Response 1.4.** The reviewer is correct that we have not undertaken an analysis that identifies statistical independence of the various phecode associations with genetically-predicted height. To do that systematically across all 142 phenome-wide significant associations (or even after grouping related phecodes) or to repeat the phenome-wide scan after systematically accounting for correlation in phecodes is beyond the scope of this manuscript. To do this would also require consideration of potential biases and correlations in electronic health record diagnosis code usage. That said, the analyses stratified by coronary heart disease status were intended to address, in a targeted way, some of these concerns regarding correlations in phecodes and statistical independence. We have tried to highlight this aspect of those analyses more clearly in the Results (lines 253-257): *“Given that CHD risk factors and CHD are correlated in our clinical data, we examined associations of genetically-predicted height with hyperlipidemia and hypertension stratifying by CHD status. That is, by stratifying by CHD status, we attempted to examine independence of genetically-predicted height associations with CHD and with hyperlipidemia, hypertension, atrial fibrillation, and venous circulatory disorders.”*

We would be willing to conduct a few additional targeted multivariable analyses that are well justified based on compelling clinical relationships/hypotheses. For the specific example given, we are unclear on the connection between atrial fibrillation and the varicose vein phenotype but perhaps the reviewer can expand on their concerns for this specific combination and any other combination of phecodes that we tested.

**Comment 1.5.** Line 153 (and elsewhere). Please specify what’s X and Y when reporting regression slopes. Do you account for estimation errors in both samples when estimating those slopes? If you then the authors could use the method developed by Qi et al. Nat Comm. 2018 (PMID 29891976)

**Response 1.5.** We appreciate this suggestion from the reviewer. For consistency with other effect size comparisons already included in the manuscript (Supplemental Figure 1), we adopt the standardized effect size Z’ approach to compare phenome-wide significant height-trait and genetically-predicted height-trait associations between race/ethnicity groups using regression slopes and correlation. The approach used in Yengo et al (2018) and Qi et al (2018) would be a suitable alternative, but we opted to use the standardized effect size comparison used in Wojcik et al (2019) since our application comparing effect sizes between race/ethnicity groups was similar to what was done in that study. The application of the method of Qi et al (2018) and Yengo et al (2018) to our context – comparing associations between samples in which we might expect differences in effect estimates (due to the height GRS being a stronger instrument in EA individuals than in AA individuals) – differs from the contexts in which the method has been applied previously for comparison of associations between two samples in which one might not expect *a priori* to find differences in the effect estimates. In other words, in our study, a regression slope ~1 would support replication of associations between race/ethnicity groups, but we would not expect a regression slope of ~1 given differences in the strength of the genetic instrument between samples. The text and reported values have been revised based on the standardized effect estimates, and a sentence describing the limitation of the regression slope and its cautious interpretation has been added:

Results (lines 171-177): “*The standardized effect estimates (Z’) for height-trait associations that reached phenome-wide significance in at least one race/ethnicity group were well correlated (r = 0.83, p = 1.4×10^-91^) between EA and AA individuals. The regression line of best fit (EA as Y ~ AA as X) between the standardized effect estimates (Z’) for height-trait associations that reached phenome-wide significance in at least one race/ethnicity group had a slope of 0.94 [95% CI 0.88, 0.99] (****Figure 1A****,* *correlation plot of unstandardized odds ratios [OR] in EA and AA).*”

Results (lines 186-200): “*The standardized effect estimates (Z’) for phenome-wide significant associations of clinical traits with genetically-predicted height were reasonably correlated between EA and AA individuals (r=0.83, p = 2.0×10^-37^) and improved when limited to traits that were at least nominally significant in AA individuals (r = 0.92, p = 1.2×10^-20^). The regression line of best fit comparing standardized effect estimates for phenome-wide significant associations of clinical traits with genetically-predicted height in EA and AA had a slope of 1.85 [95% CI 1.67, 2.02] (with EA as y ~ AA as X) and improved to 1.80 [95% CI 1.64, 1.96] when limited to traits that were at least nominally significant in AA individuals (****Figure 1B****,* *correlation plot of unstandardized OR in EA and AA). That the slope of the best fit line was >1 implies larger effect sizes in EA than in AA but such a quantitative interpretation is limited by a number of factors: differences in sample size, winner’s curse, and differences in the strength of the GRS as a genetic instrument in the two populations. An appropriately cautious interpretation would be that the regression slope and plots comparing effect sizes are consistent with concordant directions of effect for trait associations with genetically-predicted height between EA and AA individuals in MVP.* ”

Methods (lines 537-545): “*We tested concordance in direction of effect of phenome-wide significant associations of traits with measured height and with genetically-predicted height associations between EA and AA analyses using chi-square tests. For each of these pairwise comparisons, we also standardized effect estimates (beta coefficients**) to the standard error of the effect estimate and the square root of the sample size within race/ethnicity group to create a standardized Z’ effect size for each race/ethnicity group^53,54^. We then compared the standardized Z’ effect estimates for phenome-wide significant associations (with measured height and with genetically-predicted height) from EA and AA using the slope of the linear regression of beta coefficients arising from each pair of analyses being compared (e.g., height-trait associations between EA and AA).*”

**Comment 1.6.** Line 161 – 164. Note that these comparisons are affected by winner’s curse. So I’d suggest correcting estimates for that before estimating those slopes.

**Response 1.6.** We agree that accounting for winner’s curse would be important if we were using the quantitative comparison of effect sizes between race/ethnicity groups for a broader conclusion, to support claims around replication of specific trait associations with height and genetically-predicted height, or to motivate additional quantitative analyses. In this manuscript, however, the comparison of slopes is more a qualitative comparison suggesting approximate concordance between race/ethnicity groups, and we do not use the results to highlight replication of associations with specific traits between samples. We have adopted the standardized Z’ effect size to account for estimation error and sample size in this comparison as suggested in **Comment 1.5,** and added two sentences highlighting the departure of the regression slopes from 1 and a cautious interpretation of that result (see **Comment 1.5** for excerpted text).

**Comment 1.7.** Line 186. “BMI as a coefficient” => “BMI as a covariate”

**Response 1.7.** Thank you for noticing this typo; we have corrected it.

**Comment 1.8.** Line 193 (and elsewhere). Specify the unit for the that OR. Is that risk increase per height SD or per GRS SD? I’d assume the former given that the authors use a two-stage least squares approach. Could you please clarify that?

**Response 1.8.** The reviewer is correct that all of the ORs for associations with genetically-predicted height were scaled to height SD. We have clarified this when reporting ORs throughout the manuscript.

**Comment 1.9.** The diabetes analysis looks a bit artificial given that there is no statistically significant overlap between diabetes related complications and disease associated with height. The authors write (lines 179) “we noted that a number of conditions in Table 2 are associated with diabetes…”, which is no rigorous enough. So unless they can prove that there is such an enrichment, I’d suggest removing all diabetes-related sections from the main text to improve clarity.

**Response 1.9.** As two reviewers commented on clarity regarding this section, we have moved it to the online-only supplemental material. While the reviewer is correct that we did not demonstrate enrichment of diabetes-related conditions statistically, the well-described excess prevalence of diabetes in the US Veteran population (references 56-58 in the manuscript) raises concerns of confounding (less likely given weak association of height with diabetes) or effect modification by diabetes status. This relates indirectly to **Comment 1.4** in that over-represented conditions correlated with specific phecodes could induce spurious associations with genetically-predicted height in the full sample. While we did not address this limitation phenome-wide, we feel inclusion of the diabetes-related analyses addresses this concern for a number of the conditions associated with genetically-predicted height with the lowest p-values. Accordingly, we have moved it to the supplement rather than removing it from the manuscript altogether. To be consistent with this change in the main manuscript, we have removed the diabetes stratified analyses from the Abstract as well.

**Comment 1.10.** Line 308. It seems a bit odd that there could be a bi-directional causal effect between varicose veins (VV) and height given that VV is classically diagnosed in >18y. Can they authors revise that sentence (e.g., by commenting on age of onset)?

**Response 1.10.** We have revised the sentence to clarify the causal associations described in the cited paper (lines 380-382): *“These findings were further supported by a second genetic study of varicose veins that found standing height and height at age 10 were causally associated with varicose veins in MR analyses^28^.”*

**Comment 1.11.** Line 313. I think the authors should comment on why the 16 height-associated variants identified in their GWAS have not been detected before? Can they compare effect sizes and other GWAS? For example, that of Graff et al. AJHG 2021 (PMID 33713608)

**Response 1.11.** The 16 height-associated loci, represented by 24 total variants, identified in our GWAS and not in prior GWAS in European- and African-ancestry populations represent a ~2% increment in loci and <1% increment in height-associated variants compared to Yengo et al (2018). This minor variation is potentially due to chance rather than resulting from a biological or statistical reason. Given the relatively small increment in variants/loci associated with height found in our GWAS, we focused the manuscript on the MR-PheWAS. For completeness, however, we have now added information on these 24 variants from GWAS summary statistics in European- and African-ancestry individuals (Yengo et al, 2018, and Graff et al, 2021, respectively). We have added data from the summary statistics of those studies (where available) to the relevant Supplemental Tables. We found that one reason that some of the variants were not previously reported was due to differences in significance threshold – with Yengo et al (2018) using *p<*1×10^-8^ while we used 5×10^-8^. We have pointed out this discrepancy and the number of variants falling in the p-value range of 1×10^-8^ to 5×10^-8^ in the Results.

Results (lines 282-286): “*Of 22 variants at these 18 loci, all 8 that were identified in summary statistics from Yengo et al^10^ had a consistent direction of effect between MVP and the prior study (****Supplemental Table 8****). Three of the eight had p-values between* *1×10^-8^ and 5×10^-8^ so were not reported in the prior study which employed a significance threshold of p<1×10^-8^.”*

Results (lines 305-316): *“We identified 6 of these 24 variants in European-ancestry GWAS summary statistics from Yengo et al^10^ and 13 of the 24 variants in African-ancestry GWAS summary statistics from Graff et al^26^. All 6 identified in the prior European-ancestry GWAS summary statistics and all 13 identified in the prior African-ancestry GWAS summary statistics had concordant directions of effect with the multi-population GWAS results in MVP (****Supplemental Table 9****). Two of the six variants identified in prior European-ancestry GWAS summary statistics had genome-wide significant p-values in Yengo et al^10^ but were not among the 3290 variants retaining significance after the conditional and joint-multiple SNP analysis employed in that study. One of these loci – near the PIP4K2A gene – was also previously identified in a GWAS of height in Japanese individuals^27^. Taken together, potentially 14 loci, represented by 21 variants, did not have prior GWAS evidence of association with height in European- or African-ancestry individuals.”*

**Comment 1.12.** Sorry if I missed it but the authors should specify what software they have used for running GWAS. PLINK? BOLT-LMM? Also, can the authors report results from LD score regression univariate (in MVP alone) and bivariate to compare with Yengo et al.?

**Response 1.12.** Apologies for omitting the software used. GWAS analyses were performed with PLINK, and this has been added to the Methods section describing the GWAS (lines 584-586): *“Imputed and directly measured genetic variants were tested for association with the inverse normal transformed residuals of height through linear regression assuming an additive genetic model using PLINK2^59^.”*

We have also performed the requested LD score regression for the GWAS in MVP non-Hispanic White individuals, which is comparable to the Yengo et al (2018) results in European-ancestry. We have now included the numeric results for the univariate and bivariate LDSC (lines 287-297): *“**We used LD score regression (LDSC) to examine heritability and the contribution of polygenicity to global inflation in the EA GWAS and to examine genetic correlation with the most recent European-ancestry GWAS meta-analysis^10,24,25^. We found SNP-based heritability of 0.40 (standard error [SE] of 0.02) in the MVP EA sample, an LDSC intercept of 1.15 (SE 0.04), and an attenuation ratio statistic of 0.07 (SE 0.02). The LDSC intercept in the MVP GWAS of EA individuals was lower than in the most recent European-ancestry GWAS^10^. As in the prior European-ancestry GWAS, the attenuation ratio statistic from LDSC was suggestive of polygenicity contributing to a large proportion of inflated test statistics rather than population stratification. Bivariate LDSC comparing MVP EA GWAS with summary statistics from a recent European-ancestry height GWAS found high genetic correlation of 0.98 (SE 0.007) suggesting very consistent genetic effects of height between MVP EA and prior European-ancestry GWAS.”*

**Reviewer #2:** This manuscript by Raghavan et al. describes an MR-PheWAS looking for associations between height and other EHR traits. The authors use the VA Million Veterans Program (MVP) for the study, which is one of the largest datasets available with EHR data from diverse ancestry groups. Overall, this is a very good study with some interesting results.

There are a few areas of the manuscript that would benefit from additional information.

**Comment 2.1.** In line 103-105, the authors indicate that a similar study was performed in 50 traits from UK Biobank. Do any of the results from this paper replicate in UK Biobank? Were the 50 traits included in that published work included here? It does not look like the authors attempted to replicate the findings from this paper in the UK Biobank, but that seems like an obvious next step and may greatly improve the paper.

**Response 2.1.** We have not replicated the MVP MR-PheWAS in UK Biobank because the genetic risk score (GRS) that we used was based on a height GWAS meta-analysis that included the UK Biobank (Yengo et al, *Human Molecular Genetics* 2018). Applying the same GRS (derived from a GWAS that included UK Biobank) for an MR-PheWAS in UK Biobank may be prone to overfitting. Regarding comparison to the prior work, we have now added a supplemental table and a sentence comparing results from our analysis with the previous UK Biobank analysis of 50 traits that found 11 traits with concordant associations with measured and genetically-predicted height (lines 212-216): *“Of 11 traits with directionally consistent associations with measured height and genetically-predicted height previously reported in the UK Biobank^15^, 9 had analogous phecodes in MVP of which 7 replicated with the same direction of effect for the association with genetically-predicted height with at least nominal significance in MVP EA individuals (****Supplemental Table 5****).”*

**Comment 2.2.** Since the VA MVP participants are 91.6% male, did the authors consider doing a sex-stratified analysis? I am a little concerned that if there is any trait that has an unusual case distribution by sex, then the results could be confounded especially due to the drastic skew in the dataset.

**Response 2.2.** We appreciate this suggestion and have performed the suggested sex-stratified analyses with the caveat that results in women are limited by sample size. We have added the following text to the manuscript:

Results (lines 233-247): *“We repeated the MR-PheWAS after stratifying by sex within EA and AA individuals. In EA individuals, there were 1308 phecodes with ≥200 cases in men (significance p<3.8×10^-5^) and 598 phecodes with ≥200 cases in women (significance p<8.4×10^-5^). There were 135 and 6 phenome-wide significant traits associated with genetically-predicted height in EA men and women, respectively. Of the 6 phecodes associated with genetically-predicted height in EA women at phenome-wide significance, 2 were significant only in women (phecode 495, Asthma; phecode 351, Other peripheral nerve disorders), and 4 were also phenome-wide significant in men (****Supplemental Table 7****). The standardized effect estimates (Z’) for phenome-wide significant associations of clinical traits with genetically-predicted height were reasonably correlated between EA men and women (r=0.86, p = 5.4×10^-40^; best fit line slope 0.97 [95% CI 0.87, 1.08] with men as y ~ women as X). In AA individuals, there were 923 and 348 phecodes with ≥200 cases in men and women, respectively, corresponding to significance thresholds of p<5.4×10^-5^ in men and p<1.4×10^-4^ in women. We did not identify any phecodes associated with genetically-predicted height at phenome-wide significance in AA men or AA women.”*

Discussion (lines 336-340): *“Finally, we found two traits associated with genetically-predicted height in women but not in men – asthma and non-specific peripheral nerve disorders. Whether these associations reflect differences by sex in disease pathophysiology related to height may warrant exploration in a sample with better balance between men and women.”*

**Comment 2.3.** On lines 130-134, the authors describe how the sample sizes of the study participants included. Did you leave out HA because of sample size? I think it is important to state that. Also, why is the number of EA and AA lower than the first line of the paragraph? Why did people drop off?

**Response 2.3.** We have added explanations for both the change in sample size and exclusion of HA from the PheWAS analysis (lines 149-154): *“Of these, 280,451 individuals (222,300 EA and 58,151 AA) were included in the PheWAS analysis based on availability of electronic health record phenotypes needed for PheWAS.* *We did not include HA in the PheWAS due to limited sample size relative to EA and AA but the HA sample is included in an ongoing multi-biobank PheWAS effort through the GIANT consortium with a larger sample size^23^.”*

**Comment 2.4.** On line 139, the variance explained for height in the AA population is pretty low. Did you also try a meta-analysis from AA? Perhaps from this study: <https://www.sciencedirect.com/science/article/pii/S0002929721000537#mmc2>

**Response 2.4.** We appreciate the reviewer’s suggestion but have not done the meta-analysis with the Graff et al (2021) study. We have contributed GWAS data to a large international, multi-ancestry height GWAS meta-analysis that also includes most (if not all) of the data included in the Graff et al (2021) study. That multi-ancestry GWAS meta-analysis is now available online in pre-print form (Yengo et al, *biorxiv* <https://doi.org/10.1101/2022.01.07.475305>), and the sample size in non-European ancestries exceeds what we would have achieved even with a meta-analysis of our data with Graff et al (2021). In addition, we did not want to risk overfitting when estimating genetically-predicted height, so opted to use GWAS results from an independent sample from the PheWAS sample rather than using GWAS results from MVP with or without meta-analysis with Graff et al (2021). For completeness, we have now added a comparison of our multi-population meta-analysis results with the loci identified in Graff et al (2021) and Yengo et al (2018) in the Results (lines 305-316): “*We identified 6 of these 24 variants in European-ancestry GWAS summary statistics from Yengo et al^10^ and 13 of the 24 variants in African-ancestry GWAS summary statistics from Graff et al^26^. All 6 identified in the prior European-ancestry GWAS summary statistics and all 13 identified in the prior African-ancestry GWAS summary statistics had concordant directions of effect with the multi-population GWAS results in MVP (****Supplemental Table 9****). Two of the six variants identified in prior European-ancestry GWAS summary statistics had genome-wide significant p-values in Yengo et al but were not among the 3290 variants retaining significance after the conditional and joint-multiple SNP analysis employed in that study. One of these loci – near the PIP4K2A gene – was also previously identified in a GWAS of height in Japanese individuals^27^. Taken together, potentially 14 loci, represented by 21 variants, did not have prior GWAS evidence of association with height in European- or African-ancestry individuals.”*

**Comment 2.5.** On line 155-156, the authors indicate that the phewas associations were directionally concordant. If they are concordant across ancestry then how do you have EA only and AA only results listed? This is not clear. Also on lines 164-166, how is it 142 traits concordant across ancestry when only 2 were significant in AA (46 more nominally)? This is confusing.
**Response 2.5.** We realize the writing may have been unclear. The evaluation of concordance in direction of effect was not limited to traits that were significant in both race/ethnicity groups. Rather, we examined whether traits that were significant in at least one race/ethnicity groups had the same direction of effect in both groups. We have reworded both of the sentences highlighted by the reviewer in the Results section:

Lines 177-180: *“Out of 362 height-trait associations that were phenome-wide significant in at least one race/ethnicity group (212 in EA only, 133 in both AA and EA, and 17 in AA alone), 335 had concordant directions of effect between the two race/ethnicity groups (p < 2×10^-16^).”*

Lines 200-203: *“Though only 48 out of 142 traits associated at phenome-wide significance with genetically-predicted height in EA were at least nominally significant in AA, 124 out of those 142 traits had the same direction of effect in EA and AA for association with genetically-predicted height (p = 4×10^-16^).”*

**Comment 2.6.** On line 170, the authors indicate that 2 traits are significant in AA. The number of significant traits is substantially different between ancestry groups. Any idea what is driving this? Is it power and sample size? Is it differences in health care utilization with different volumes of EHR data and diagnoses? Is it because the AA group is much younger so less diagnoses? Or is it biology?
**Response 2.6.** This is an important question that is difficult to answer without speculation. To partially address this, we examined the number of EHR-derived phecodes associated with EA and AA individuals and found that they were similar (46±33 phecodes in EA and 48±32 phecodes in AA). While the mean number of phecodes associated with individuals was similar, it’s possible that age differences in the samples of EA and AA individuals in our study reflect differential accumulation of phecodes associated with genetically-predicted height. However, given the similarity in phecode burden between the two race/ethnicity groups, we think it is more likely that the differences in the number of significant traits results from a combination of the discrepancy in sample size and quality of the genetic instrument for height (i.e., the height GRS is a better genetic instrument in EA than in AA given that it is derived from a European-ancestry GWAS). While we had included the difference in quality of the genetic instrument between race/ethnicity groups among the limitations, we did not explicitly acknowledge the difference in sample size as a limitation.

We have now added the discrepancy in sample size as a limitation in the Discussion (lines 410-416): “*Second, we had a substantial discrepancy in sample size between EA and AA individuals in MVP which undoubtedly contributed to the variation in the number of traits associated with genetically-predicted height at phenome-wide significance between the two race/ethnicity groups. As has been recognized for GWAS, only with expansion of non-European ancestry and non-White race/ethnicity samples will we be able to determine if phenome-wide associations of traits with genetically-predicted height are common across or vary between ancestry and race/ethnicity groups.*”

We have also added the burden of phecodes by race/ethnicity group to the Results (lines 154-155): “*The electronic health records of EA and AA individuals reflected similar numbers of phecodes: 46±33 phecodes in EA and 48±32 phecodes in AA (mean±SD).*”

**Comment 2.7.** The relationship between diabetes and height is not totally clear at the end of the section ending on line 212. Perhaps this section needs a summary at the end.

**Response 2.7.** Reviewer 1 also had concerns about the diabetes analyses (see **Comment 1.9** above), so we have moved this section to the online supplemental material. Many of the phecodes associated with genetically-predicted height with the lowest p-values are conditions associated with diabetes, and diabetes is more prevalent in Veterans than in the general US population. Therefore, we felt that examining whether diabetes status modified the MR-PheWAS associations with these selected traits addressed an important potential source of bias even though genetically-predicted height was only weakly and inversely associated with diabetes status. We have added some clarifying language to the text that now appears in the supplement to describe these analyses.

**Comment 2.8.** On line 420, did case status apply to individuals with one instance of the ICD code or did you require a rule of 2 or 3?
**Response 2.8.** Case status for phecodes required the use of 2 related ICD codes. We have added this detail to the Methods (lines 497-499).

**Comment 2.9.** On line 436, How did you decide on 10 PCs? How much variance was explained by these?

**Response 2.9.** We included 10 PCs as convention for adequately accounting for population structure (Price et al, *Nature Genetics* 2006). In the MVP data, 10 PCs have been previously used when developing the HARE algorithm, including for a demonstration of the HARE algorithm to a GWAS of height (Fang et al, *AJHG* 2019). We have added these citations to the indicated line in the Methods section.

**Comment 2.10.** Line 128, the abbreviation "HA" was not yet defined.

**Response 2.10.** Thank you for noticing this- we’ve expanded the text at this first use of the abbreviation.

**Comment 2.11.** Line 137, the abbreviation "GRS" was not yet defined.

**Response 2.11.** Thank you for noticing this- we’ve expanded the text at this first use of the abbreviation.

**Comment 2.12.** Throughout the paper, the authors use race/ethnicity to define the groups, however using HARE, they are also using ancestry. I wonder if the word "ancestry" should be used? Or perhaps race/ethnicity/ancestry? They mean different things of course. But since genetics was used to define, I think it is important to include ancestry in the paper. Similarly, should it be "trans-ancestry" or "cross-ancestry"?

**Response 2.12.** This is a difficult question and one that we’ve discussed at length in the core writing group. Because HARE trains a model to use genetic information to predict self-identified race/ethnicity, the HARE-based classification assigns individuals into race/ethnicity groups rather than ancestry groups. While there is certainly overlap between the HARE race/ethnicity groups and continental ancestry groups, we limit the terminology in the manuscript to race/ethnicity since that captures the classification outcome of HARE most accurately and describe the study using the term “multi-population” as suggested in a recent commentary in *Nature Genetics* (Kamariza et al, PMID 34741159). To address this, we have added the following to the Methods (lines 504-508): “*As the HARE algorithm uses genetic data to predict race/ethnicity^50^, we describe the populations studied here as race/ethnicity groups rather than ancestry groups and describe the study as a multi-population analysis rather than multi-ancestry. Accordingly, we classified individuals as non-Hispanic White (EA), non-Hispanic Black (AA), and Hispanic-American (HA) using HARE.”*

For consistency, we’ve also modified the terminology from trans-ethnic to multi-population as most of the analyses were performed in parallel between race/ethnicity groups.

**Comment 2.13.** On line 198, there is something odd with reference.
**Response 2.13.** Thank you for noticing this issue. The relevant section of text has been moved to the online supplemental material, and we’ve corrected the citation formatting there.

**Comment 2.14.** What software was used for the GWAS?

**Response 2.14.** We appreciate the reviewer catching this oversight. We used PLINK and have added this to the Methods (lines 584-586): “*“Imputed and directly measured genetic variants were tested for association with the inverse normal transformed residuals of height through linear regression assuming an additive genetic model using PLINK2^59^.”*

**Reviewer 3:** In reading “A multi-ancestry phenome-wide association study of genetically-predicted height in the Million Veteran Program” by Raghavan and colleagues, I honestly can not tell if I am having some sort of moment of senility, the paper literally forgets to present any of the results it claims, or if it is merely poorly written in a way that makes it exceptionally challenging to understand. There is so much of this paper that makes no sense to me, it is hard to figure out where to start. The simplest place, probably begins with saying, as far as I can tell the authors have not done any Mendelian Randomization analysis. If any MR was done, it is not described or presented in the paper in any recognizable way. In fact as far as I can tell, the only analysis the authors have done is to replicate the problem Mendelian analysis aims to solve. Beyond this central issue with analysis, there is also a fundamental question of why and how are the authors presenting data from both EA and AA individuals.

**Comment 3.1.** I have no idea what the authors mean by the words “Mendelian randomization.” Nothing about this analysis is an MR in any sense that I understand those terms. We start with the obvious, if A is correlated with B, and B is correlated with C, it is not surprising to find that A is correlated with C. We have learned nothing from asking the question “Yes or no: Is A correlated with C, given that we already know that A is correlated with B and B with C?” As far as I can tell that is all the authors have done here. They show that a genetic risk score (GRS) for height is correlated with measured height. They show that measured height is associated with some other trait T. They sometimes, but not always, show that the GRS for height is associated with trait T. So, why do I care? A (GRS) is associated with B (Height), B (Height) with C (Trait), and now you establish that some of the time A (GRS) is associated with C (Trait). That one of those things is a measure involving genotypes (GRS) does not make this a Mendelian randomization.

**Response 3.1.** We used a two-stage least squares Mendelian randomization design which does not simply examine association of A (GRS) with C (Trait). Rather, it uses the association of A (GRS) with B (Height) to estimate genetically-predicted height for each participant, then examines the association of that genetically-predicted height with traits (see Sanderson et al, *Int J Epidemiology* 2019, PMID 30535378; and Burgess et al, *Stat Methods Med Res* 2017, PMID 26282889). We agree that evaluating the association of a GRS with traits would not represent a Mendelian randomization study. The genetically-predicted height captures only the extent to which an individual’s height is explained by genetic variation, which is less susceptible to confounding than measured height. In that sense, this approach uses a GRS as an instrumental variable for height – the principle of a Mendelian randomization study. In addition to the above references that describe the Mendelian randomization method used in our study, the first MR-PheWAS study that uses Mendelian randomization methods in a phenome-wide association study framework employed a two-stage least squares design in their genetic instrumental variable analysis similar to what we have used in this study (Millard et al, *Sci Reports* 2015, PMID 26568383).

**Comment 3.2.** In a classical Mendelian randomization we are trying to show something (B) is not merely correlated with something else (C), but causally related in the sense that changing B causes changes in C, rather than both B and C sometimes change at the same time in the same direction. To attempt to establish such a causal relationship, one can look for a third trait, A, that has the property that it is necessarily unaffected by B and C (thus, changing B or C during the course of a lifetime cannot alter A), and that A is correlated with both B and C. To test

whether data is consistent with the hypothesis that B causes C, and that A does not, other than via its effects on B, we can ask whether the relationship between A and B, and B and C, fully explains the apparent relationship between A and C. If it does, than the data is consistent with

the idea that B causes C, i.e. that B is a causal risk factor for C.

In this particular case, B is height. We wish to know if height is a causal risk factor for any other these other traits. Here A is genotype. One is born with genotype, and genotype is not modified by height or any other trait. The authors start be establishing that genotype (GRS, A) is correlated with measured height (B). The authors then establish that measured height (B) is associated some other trait T (C). Finally the authors establish that at least some of the time GRS is associated with that same T. Thus, the authors establish that have sufficient data to do an MR experiment… And then….. did I miss it? Is there some supplement somewhere? I looked, and looked, and thought I was losing my mind, but as far as I can tell there is no MR experimental data anywhere in this paper?!?!? They establish that one might do such as test…but then don’t.

**Response 3.2.** Based on the reviewer’s comments, we have tried to be more explicit in the Methods section about each of the steps in the analysis. To clarify, the third step that the reviewer describes (“Finally the authors establish that at least some of the time GRS is associated with that same T”) is where our approach differs from his description. The final step in the two-stage least squares method is to evaluate association of genetically-predicted height rather than the GRS with traits. The genetically-predicted height uses the first stage regression of height on the GRS to estimate height determined by genetic variation; this differs from an association of the GRS itself with phenome-wide traits. We have revised the Methods as follows (lines 514-525, lines 533-536): *“In the MR-PheWAS, associations of EHR traits with genetically-estimated height were performed using a two-stage least squares regression^13,21^. In the first stage, we performed race/ethnicity-stratified linear regression of measured height (dependent variable) on the height GRS, adjusting for 10 race/ethnicity-specific PCs. Next, we estimated genetically-predicted height for each participant using the first-stage regression equation, and standardized the resulting genetically-predicted height for each participant to the standard deviation of measured height. Thus, a one-unit change in the standardized genetically-predicted height corresponds to a one-unit change in the height Z-score. In the second stage, we used the R PheWAS package^31^ to perform a race/ethnicity-stratified MR-PheWAS. This package uses logistic regression to estimate associations of phecodes as the dependent variables and the standardized genetically-predicted height estimated from the first stage as the independent variable, adjusting for age at enrollment in MVP, sex, and 10 race/ethnicity-specific PCs. .... A significant association in this second-stage of the MR-PheWAS suggests an association of the phecode with variation in height due to genetic variants not merely an association of the phecode with measured height or with the GRS.”*

Regarding the following comment from the reviewer “To test whether data is consistent with the hypothesis that B causes C, and that A does not, other than via its effects on B, we can ask whether the relationship between A and B, and B and C, fully explains the apparent relationship between A and C. If it does, than the data is consistent with the idea that B causes C, i.e. that B is a causal risk factor for C”, this appears to describe validation of the so-called exclusion restriction assumption of Mendelian randomization (and all instrumental variable analyses) that the association of the instrument (A) with the outcome (C) occurs solely through the exposure of interest (B). However, a causal association of B with C can exist even when there is an association of A with C not fully explained by the relationships of A with B and B with C – for example, in the presence of pleiotropic effects. Moreover, the exclusion restriction assumption cannot be verified in most, if not all, scenarios. The two-stage approach we used evaluates whether there are associations of the predicted value of B (height) based on A (GRS) with traits (C) rather than the association of A with C. This does not require that there is no relationship of A with C independent of B, but can still provide information about putative causality in associations of B with C. Please also note that we do not make any claims regarding the magnitude of the causal effects of height with phenome-wide traits beyond comparing effect estimates between race/ethnicity groups.

**Comment 3.3.** There are surely many ways to do an MR type experiment with this data, but the simplest would be use the Beta from the regression of GRS on Trait (Beta1) as the outcome (Y), and ask to what extent is Beta1 accounted for by the relationship between GRS on measured height (beta2), and measured height on Trait (beta3). To do this, for every sample in my “million” study, I would use the GRS prediction for trait (beta1) as my outcome Y. I would then use GRS to predict height (beta1), and then use the predicted height as it were the measured height, by plugging estimated height into the relationship between measured height and trait (beta3), an use this as my predictor. Thus, I am regression predicted trait estimated by GRS on height and then height on trait, against GRS on trait. If that regression is identically 1, it would strongly establish that genotype’s correlation with the trait is almost surely being explained by genotype’s effects on height, and heights causal effect on trait, and I would thus conclude that height is a causal risk factor for trait. In fact, any statistically significantly positive relationship between trait predicted from GRS(beta1) and trait predicted from GRS by way of height on trait (beta2*beta3) would tend to be taken as some sort of evidence for at least a partially causal role height on trait.

**Response 3.3.** We agree with the reviewer that the proposed approach is one way to perform an MR with a single study sample. The two-stage least squares approach has parallels to what the reviewer proposed in that it tests whether the estimate for trait predicted from GRS by way of height (i.e., using genetically-predicted height, not the GRS itself) differs from null (zero). It does not compare that value to the association of trait predicted by the GRS itself, but the latter comparison is not required to infer causality between height and trait. Finally, we reiterate that in the context of our phenome-wide study, we did not perform systematic evaluations for pleiotropy which could yield associations from the MR analysis in the absence of a causal relationship of height with traits (lines 416-421 in the Discussion): *“Third, we did not interrogate genetic correlation or pleiotropy between height and associated traits identified in the MR-PheWAS. To perform such analyses on a phenome-wide scale exceeds the scope of this manuscript. Thus, we are cautious about causal interpretation of the results of the MR-PheWAS reported here in the absence of such secondary analyses. Given increasing numbers of clinical biobanks globally, replication of MR-PheWAS associations in independent cohorts will be the focus of future work.”*

**Comment 3.4.** Okay, so the authors need to do some MR analysis if they want to claim they have learned anything more than GRS predicts height, height predicts other traits, and thus, not surprisingly, at least some of the time GRS predicts traits. If the authors do an MR analysis with this data it could be interesting, but they also need to do something about how / why they are presenting data from both EA and AA populations. At no point could I figure out if I was to supposed think about one as a “discovery cohort” and the other as “replication” (and if so which is which), or was this fundamentally two “independent” studies asking the same questions in both studies. If I was to understand it as the latter, why were all the result presented an intertwined, simultaneous manner between the two, completely breaking the flow of the narrative in either population, and making it far, far harder to keep track of what result was what. If these are independent studies, present one, and then the other, and discuss similarities / differences and why you think that is afterwards. On the other hand, if I am supposed to view one of these as a discovery cohort, and the other as replication, you need to be much more explicit about what is, and isn’t considered replication, and which results actually did replicate. At first blush it sort of appears that almost “nothing” replicated, but that might not be surprising given the differences in sample size. Frankly if these are supposed to be thought of as independent experiments I might even consider breaking them into two papers, or at the very least, two very distinguishable subsets of the same paper.

**Response 3.4.** The reviewer proposes an alternative structure to the manuscript, which has merit but is inconsistent with our intentions. We do not describe the two race/ethnicity groups as discovery and replication cohorts because they were not investigated in that manner. Moreover, using them in that way would raise several concerns regarding validity of stratification by race/ethnicity for the purposes of split discovery and replication samples. The alternative, which the reviewer mentions, was our intent: to perform parallel analyses in two samples that were stratified based on race/ethnicity. We have organized the Results by analysis rather than by race/ethnicity with the goal of comparing results between race/ethnicity within each section of the Results. While multi-ancestry GWAS have been historically limited until recently, this organization of Results – describing parallel analyses in each sample then comparing them and/or meta-analyzing (which we’ve added to in the revised manuscript) – is not uncommon. We also believe there is value in directly comparing results in disparate race/ethnicity or ancestry groups given that such analyses have historically not been performed in parallel or have prioritized European ancestry samples. While the direct comparison of results between the two race/ethnicity groups in our MR-PheWAS is limited by the sample size difference, as remarked by the reviewer, the comparison highlights similarities in associations phenome-wide even if not significant in the smaller sample. In response to the detailed constructive comments from all reviewers, we believe the manuscript organization and clarity has improved through revision.
